# Important role of indels in somatic mutations of human cancer genes

**DOI:** 10.1186/1471-2350-11-128

**Published:** 2010-09-01

**Authors:** Haiwang Yang, Yan Zhong, Cheng Peng, Jian-Qun Chen, Dacheng Tian

**Affiliations:** 1 State Key Laboratory of Pharmaceutical Biotechnology, Department of Biology, Nanjing University, Nanjing 210093, China

## Abstract

**Background:**

Cancer is clonal proliferation that arises owing to mutations in a subset of genes that confer growth advantage. More and more cancer related genes are found to have accumulated somatic mutations. However, little has been reported about mutational patterns of insertions/deletions (indels) in these genes.

**Results:**

We analyzed indels' abundance and distribution, the relative ratio between indels and somatic base substitutions and the association between those two forms of mutations in a large number of somatic mutations in the Catalogue of Somatic Mutations in Cancer database. We found a strong correlation between indels and base substitutions in cancer-related genes and showed that they tend to concentrate at the same locus in the coding sequences within the same samples. More importantly, a much higher proportion of indels were observed in somatic mutations, as compared to meiotic ones. Furthermore, our analysis demonstrated a great diversity of indels at some loci of cancer-related genes. Particularly in the genes with abundant mutations, the proportion of 3n indels in oncogenes is 7.9 times higher than that in tumor suppressor genes.

**Conclusions:**

There are three distinct patterns of indel distribution in somatic mutations: high proportion, great abundance and non-random distribution. Because of the great influence of indels on gene function (e.g., the effect of frameshift mutation), these patterns indicate that indels are frequently under positive selection and can often be the 'driver mutations' in oncogenesis. Such driver forces can better explain why much less frameshift mutations are in oncogenes while much more in tumor suppressor genes, because of their different function in oncogenesis. These findings contribute to our understanding of mutational patterns and the relationship between indels and cancer.

## Background

Humans are increasingly exposed to food-, water- and air-borne carcinogens, as well as specific carcinogenic agents related to their occupational settings and life-style choices [1,2]. Indeed, cancer is responsible for more than one-fifth of all deaths worldwide [3] and can be characterized by such hallmarks as uncontrollable growth, immortality and metastasis, and induction of inflammatory microenvironment [4]. Accumulation of genetic changes can give rise to tumorigenesis in three types of genes: oncogenes, tumor-suppressor genes and stability genes [5,6]. Cancer-related genes are those which are mutated and are causally implicated in cancer development [7]. Unlike certain other diseases which can be caused by mutations in one specific gene, human cells have many safeguards to protect itself against the lethal effects of cancer causing genetic mutations [5]. Therefore, it is the defectiveness of a subset of genes that give rise to the development of cancer [8], and many DNA changes in cancer genes can contribute to the final state of tumorigenesis. Moreover, the accumulation of somatic mutations in oncogenes and tumor suppressor genes is thought to play a critical role in cancer development and leads to the multi-step conversion of normal cells to a malignant state [9-11].

All cancers arise from the DNA sequence changes of the cellular genomes. However, not all changes directly lead to the development of cancer; instead, cancer is caused by a series of genetic alterations accumulated in the key tumor-suppressor genes and oncogenes [6,12]. A large number of cancer genes have been identified through decades of studies, and the pace will be raised with the coming of new revolutionary sequencing technologies. The Catalog of Somatic Mutations in Cancer (COSMIC database), a large-scale database founded by the Wellcome Trust Sanger Institute, is designed mainly to store and catalog somatic mutation information with regard to human cancers. A vast amount of published somatic mutations involved in cancer genes has been gathered in the COSMIC database [13]. The mutated genes are always oncogenes and tumor suppressors that are involved in the generic processes of cell-cycle control, signal transduction, and stress responses [8]. COSMIC was initiated in 2004 and presently is the most comprehensive resource for information on somatic mutations in human cancers, providing over 50,000 mutations in almost 4800 cancer genes for investigation [13,14]. The data can also be queried by sample, which allows us to analyze different mutations occurring within the same genome.

The most commonly observed somatic mutation in cancer-related genes includes base substitution and insertion/deletion (indel) [7,15]. In COSMIC, most of the mutations are in these two forms. Previous reports observed a co-variation of base substitutions and indels [16-20] as well as their non-random distribution which generate mutation hotspots in certain human disease loci [21-23]. Recently, the nucleotide substitution rate was found to be significantly elevated surrounding indels and correlated with both indel size and abundance [24], suggesting an important role played by indels as well in the mutations of cancer-related genes. To test this hypothesis, we investigated the distribution and abundance of both indels and somatic base substitutions. We found that the indel-centered distribution of base substitution is a general pattern and that indels are particularly abundant in somatic mutations of cancer-related genes. Our finding may shed light on the mechanisms of how cancer-related mutations arise and provide further evidence for future directions of study on such mechanisms.

## Methods

### Data source of mutations and sequences

All somatic mutational data were retrieved from COSMIC (v47 release; http://www.sanger.ac.uk/genetics/CGP/cosmic/). The genomic sequence and annotation information from the human-Ref and chimpanzee genomes were obtained from the Ensembl 52 database http://www.ensembl.org. The human-Celera and human-Korean sequences were retrieved from the ftp site at the NCBI ftp://ftp.ncbi.nlm.nih.gov/genomes/H_sapiens/ and the publication by Kim et al. 2009, respectively [25]. The homologous relationships of the cancer genes in different genomes were confirmed by protein similarities obtained from BLASTp searches, except for human-Korean, whose homologous relationship was already provided based on human-Ref. Peptide sequences of the cancer genes were retrieved from the COSMIC FTP site ftp://ftp.sanger.ac.uk/pub/CGP/cosmic/fasta_files/.

### Analysis of indels and base substitutions in the coding regions of cancer genes

A gene in the COSMIC database is represented by a single transcript for a given gene, and the cDNA in COSMIC refers to the coding sequence (CDS) [13]. Only the unambiguous mutation data with definite position and base changes in COSMIC were included in our analysis. Twenty-five cancer genes with ≥100 mutations (either indels or somatic base substitutions) in their CDSs were used to analyze the co-localization of indels and base substitutions. Each CDS was equally divided into 10 blocks, and the densities of indels and base substitutions were calculated in each block. We considered a cancer gene as an apparent mutation bias ("apparent" category in Table 1) if it reaches the three criteria: a) the top three blocks with the most indels contain 60% of all indels; b) the top three blocks with the most substitutions contain 60% of all substitutions; c) at least one block is shared by both indel and substitutions. To further explore the co-localization of indels and base substitutions, the indel number in each block was plotted against their corresponding substitution number. The co-localization was considered significant ("significant" category in Table 1) when R^2 ^> 0.40 and *P *< 0.05 by Pearson's correlation. Finally, genes that do not reach the significance criteria were deemed as insignificant.

**Table 1:** Statistics of cancer genes with not less than 100 mutations.

Gene name	Total mutation number	**Number of base substitutions**^**a**^	Number of indels	S/I ratio	Category of S-I linkage	Gene type
APC	808	266 (11, 255)	542	0.49	Apparent	Tumor suppressor gene
PTEN	769	407 (19, 388)	362	1.12	Insignificant	Tumor suppressor gene
VHL	756	296 (38, 258)	460	0.64	Significant	Tumor suppressor gene
CDKN2A	457	302 (57, 245)	155	1.95	Insignificant	Tumor suppressor gene
NF2	385	80 (5, 75)	305	0.26	Significant	Tumor suppressor gene
CEBPA	358	43 (8, 35)	315	0.14	Insignificant	Other
KIT	312	148 (25, 123)	164	0.90	Apparent	Oncogene
TP53	300	232 (5, 227)	68	3.41	Significant	Tumor suppressor gene
EGFR	269	208 (19, 189)	61	3.41	Apparent	Oncogene
CTNNB1	229	143 (17, 126)	86	1.66	Apparent	Oncogene
NOTCH1	222	98 (9, 89)	124	0.79	Apparent	Tumor suppressor gene
PIK3CA	204	190 (9, 181)	14	13.57	Apparent	Oncogene
PTCH1	189	125 (10, 115)	64	1.95	Insignificant	Tumor suppressor gene
NF1	182	83 (5, 78)	99	0.84	Insignificant	Tumor suppressor gene
RB1	174	87 (4, 83)	87	1.00	Significant	Tumor suppressor gene
ATM	162	124 (2, 122)	38	3.26	Insignificant	Other
WT1	156	44 (0, 44)	112	0.39	Apparent	Tumor suppressor gene
SMAD4	147	108 (4, 104)	39	2.77	Insignificant	Tumor suppressor gene
RUNX1	135	60 (6, 54)	75	0.80	Apparent	Tumor suppressor gene
CDH1	133	70 (6, 64)	63	1.11	Insignificant	Tumor suppressor gene
FLT3	124	35 (4, 31)	89	0.39	Apparent	Oncogene
MEN1	122	45 (1, 44)	77	0.58	Insignificant	Tumor suppressor gene
BRAF	118	109 (16, 93)	9	12.11	Apparent	Oncogene
STK11	111	66 (8, 58)	45	1.47	Significant	Tumor suppressor gene
GATA1	106	20 (2, 18)	86	0.23	Apparent	Other

***Mean***	277	136 (12, 124)	142	0.96	-	-

S/I is the ratio of base substitution number and indel number. S-I represents the linkage between substitutions and indels. In column *a*, the two numbers in parentheses are synonymous and non-synonymous somatic base substitutions, respectively. Gene type is "Oncogene" or "Tumor suppressor gene" whenever there is strong evidence according to previous publications (otherwise "Other" is assigned).

### Analysis of the distance between indel and base substitution within the same samples

In the COSMIC database, any given sample may have more than one mutation, which means a series of mutations may occur within the same sample. Using a Perl program, we obtained 511 samples with at least one indel and one base substitution, and analyzed the distribution of the distances between indel and its nearest base substitution. To obtain a general view of this distribution, we analyzed the data at the 20 bp and 1 bp scale within 300 bp and 20 bp regions, respectively.

### Evolutionary analysis of the genomic region of cancer genes

The peptide sequences of cancer genes were used as a query in BLASTp searches against proteins of other genomes. The threshold of expectation value (E-value) was set to 10^-4^. The protein hits with the smallest E-value were selected, and their corresponding genomic gene regions were retrieved by a Perl program. The Mauve program http://asap.ahabs.wisc.edu/mauve/ was used to align the genomic sequence, and a Perl program was used to obtain alignments that are >1000 bp. For each indel that is <100 bp in length, the 5' and 3' flanking sequences were divided into five 100 bp windows, when both 5' and 3' flanking sequences are longer than 500 bp. We also validated the homologies by eliminating the alignments with any blocks that have >10 mutations.

## Results

### Relative abundance of indels and base substitutions in cancer genes

The COSMIC database contains non-redundant somatic mutations, including samples which have been found to be negative for mutations during screening in different genes of different cancer types [26]. Therefore, this data enables a comprehensive mutational frequency analysis.

**Figure 1:**
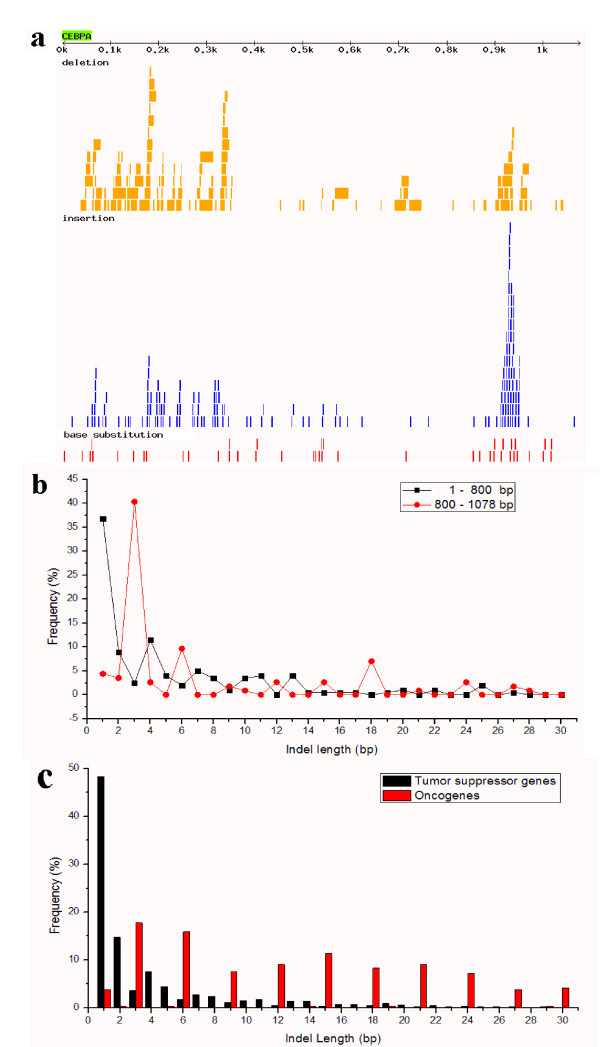
**Abundance and size distribution of indels in the gene CEBPA and in oncogenes/tumor suppressor genes**. (a) Mutational pattern in the CDS of CEBPA; (b) comparison of indel size distribution between 1 - 800 bp and 800 - 1078 bp region in CEBPA; (c) comparison of indel size distribution between oncogenes and tumor suppressor genes identified in Table 1. To make indels as less overlap as possible, only ≤30 bp indels are shown in (a). Only ≤30 bp indels are shown in (b) and (c).

In this database, one of the most significant characteristics of cancer-related mutations is the high frequency of indels, as compared to that of somatic base substitutions. In general, indels constitute about 27% of all non-redundant mutations in COSMIC database. The proportion is even higher in the 25 genes each with ≥100 mutations that we chose to study in depth (Table 1 and Additional file 1: Supplemental Fig. S1); among these genes, the indels take up about half of all mutations. Notably, the proportion of indels varies drastically among different cancer genes. In the PIK3CA gene, for example, base substitution is about 14 times more than indels (Table 1). By contrast, more than 7/8 of the mutations in the CEBPA gene are indels (Table 1). The indels in CEBPA also display diverse types and multiple lengths, particularly in the 3' ends of the CDS (Fig. 1a &1b). The indel sizes at the hot-spot (e.g., the 800 - 1078 bp region) are far from random. In this region, the indel size ranges from 1 to 181 bp in the total of 114 indels. More surprisingly, 85% (97 out of the 114) indels are 3n size multiples, and there are only 9 indels <3bp. This distribution is significantly different from that of the 1 - 800 bp of the CDS in CEBPA (Fig. 1b; Chi-square test for 1 - 15 bp data, *P *< 0.01) and also the overall indel sizes of all cancer genes (Additional file 1: Supplemental Fig. S2; Chi-square test for 1 - 15 bp data, *P *< 0.01), in which 1 - 2 bp indels account for most of the indels.

The ratio of nucleotide substitutions to indels (S/I) between human genome sequences is 11.39 within coding regions [27]. This ratio should reflect the abundance of substitutions and indels in meiotic mutations. However, the COSMIC database contains mostly missense changes [28], suggesting that the calculated substitution/indel ratios (S/I in Table 1) might reflect their relative importance in causing the cancer cell phenotypes. Indeed, in COSMIC, the average ratio is far less than 11.39, indicating an important role played in by indels. Interestingly, the indels are more abundant in the genes with rich somatic mutations. For example, the overall S/I ratio is 2.66 for all the 4408 genes with at least one mutation (indel or base substitution); 1.17 for all the 251 genes with at least one indel and one base substitution; and 0.96 for all the 25 genes with ≥100 mutations. Only in two out of the 25 genes (Table 1), the S/I ratio is larger than 11.39. A similar trend is also observed for non-synonymous substitutions, which are 10 times more abundant than synonymous substitutions in the genes with ≥100 mutations (Table 1), as compared to a ratio of 4.5 in the overall COSMIC data.

For tumor suppressor genes, loss of function is associated with oncogenesis, in contrast to the gain-of-function activation of oncogenes [29]. In this scenario, one might expect that the non-3n indels, to frequently produce frameshifts, might be more frequent than the other genes (e.g., oncogenes). In Table 1, 16 tumor suppressor genes and six oncogenes are identified, which contain 88% and 5% of non-3n indels, respectively. The proportion of non-3n indels is significantly different between these two gene types (Fig. 1c; Chi-square test for 1 - 30 bp data, *P *< 0.01). The tumor suppressor genes indeed produce more frameshift mutations than oncogenes.

### Co-localization of indels and base substitutions in cancer gene CDS

**Figure 2:**
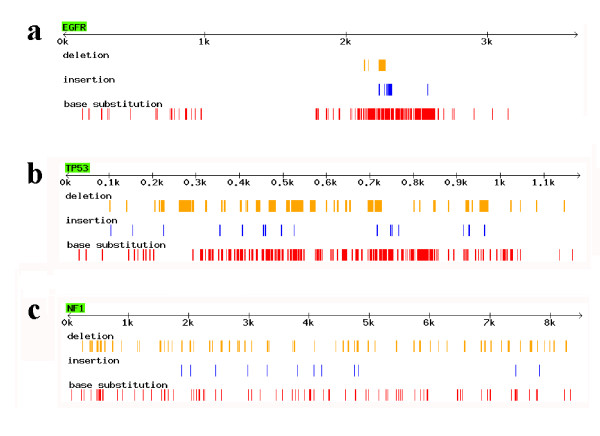
**Examples of the genes in 'apparent', 'significant', and 'insignificant' categories**. (a) Mutational pattern in EGFR as an example of the 'apparent' category (for detailed definition, see Methods); (b) in TP53 as an example of the 'significant' category; (c) in NF1 as an example of the 'in significant' category. Gene names are shown in green boxes. The Arrow below the gene name denotes position in the CDS, and 'k' means 1000 bp length of DNA. To make indels as less overlap as possible, only ≤30 bp indels are shown.

In order to uncover the mutational patterns of indels and base substitutions in human cancer genes from COSMIC, 25 cancer genes with ≥100 mutations, either in the form of indel or base substitution, were analyzed. They were then classified into three categories ('apparent', 'significant' and 'insignificant' in Table 1) according to the extent of co-localization between indel and base substitution (detailed criterion is described in Methods). To illustrate the distribution of indels and base substitutions, the mutation data was visualized using a Perl program (comparison of the three categories is shown in Fig 2).

**Figure 3:**
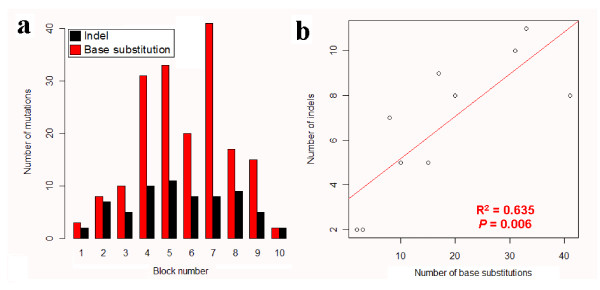
**Illustration of significant correlation between indel and base substitution in ten-block analysis**. (a) Number of mutations in ten sequential blocks of TP53; (b) scatter plot of indels and base substitutions in TP53; Graphic view of mutations in TP53 is in Fig. 2b.

The co-localization of indel and base substitution is visually 'apparent' when the first category of genes is shown graphically (e.g., EGFR in Fig. 2a), where the indels and substitutions are highly biased towards a certain position in a CDS. In total, 11 'apparent' genes are identified and their mutational distributions are shown in Additional file 1: Supplemental Fig. S3. For the other 14 genes, however, the correlation might not be graphically observed, due to the fact that so many mutations occurred along the CDS (for example, the genes TP53 and NF1 in Fig. 2b &2c). When the indels and base substitutions were calculated separately in the equally divided 10 blocks of CDSs, some genes showed close correlations between these two types of mutations (Fig. 3 and Additional file 1: Supplemental Fig. S4). Five genes are classified into the second category with a 'significant' correlation, based on a R^2 ^> 0.40 and *P *< 0.05. Notably, for the genes in the 'apparent' category, eight out of the 11 genes are also qualified well under the 'significant' criteria (Additional file 1: Supplemental Fig. S4). However, we did not put them in the 'significant' category because their mutations are sometimes so biased that in certain blocks there is no mutation data (e.g., CTNNB1 in Additional file 1: Supplemental Fig. S4), making it difficult to evaluate the validity of significance.

The remaining nine genes have no significant associations between indels and substitutions and are therefore classified into the 'insignificant' group (for example, NF1 in Additional file 1: Supplemental Fig. S5; Additional file 1: Supplemental Fig. S4). However, when shown in a detailed illustration, 4 out of the 9 genes (CDKN2A, CEBPA, SMAD4 and MEN1) have their R-square above or close to 0.30 (Additional file 1: Supplemental Fig. S4), indicating a minor co-localization of indel and base substitution. Furthermore, some of them displayed a clear linkage between these two types of mutations (i.e., CEBPA in Fig. 1a). Therefore, we conclude that for at least 16 out of the 25 genes (11 'apparent' and 5 'significant'), indels and substitutions indeed tend to co-localize in the CDS or at least correlate strongly with each other.

Additionally, to confirm its validity of our 'three-category' approach, we also explored all the 10 cancer genes with 50 - 99 mutations, in which a highly consistent result was obtained (Additional file 1: Supplemental Fig. S6). Although the approach is not designed to work perfectly for genes with few mutations, we still found six out of the ten genes reach the 'apparent' criteria, and three (KRAS, NPM1, JAK2) of those genes reached the significant criteria. This indicates that the pattern observed in the genes with ≥100 mutations is common among cancer-related genes.

### Associations of indels and base substitutions within the same sample

**Figure 4:**
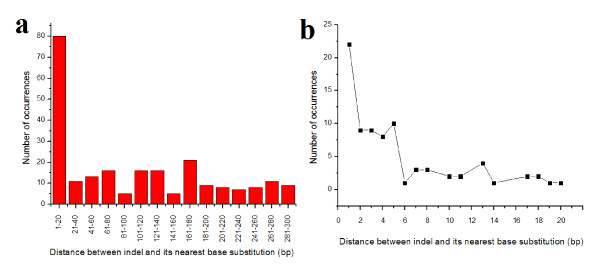
**Distribution of the distances between indel and the nearest base substitution in the same sample**. (a) Distribution of the distances in 20 bp scale analysis and only the distances within 300 bp are shown; (b) Distribution of the distances in one bp scale analysis and only the distances within 20 bp are shown.

The results above indicate that in cancer-related genes indels and base substitution tend to accumulate in the same region within the CDS. This finding was based on the analysis of the non-redundant mutational data in cancer-related genes with ≥100 mutations. To gain another perspective, we also analyzed samples each with both indels and base substitutions in order to explore their co-occurrence in a more direct manner. In the COSMIC database, it is possible to analyze multiple mutations that occurred in the same cancerous sample, and it was our goal to study in these cases the distribution of distance between indels and its nearest base substitutions. Samples with at least one indel and one base substitution were analyzed. Data from 511 samples with regard to 48 cancer genes were collected. The distance distribution between indel and substitution is shown in Fig. 4a, and the number of occurrences is concentrated in the 1-20 bp range, indicating that indels and base substitutions indeed tend to occur closely to each other. A detailed illustration of the distance distribution between 1 bp and 20 bp is shown in Fig. 4b, and the correlation is significant (R-square = 0.83 and *P *< 0.01) when fitted by an exponential decay curve. In both large and small scale analyses (Fig. 4), the number of occurrences with short distances between the indel and substitution were higher than those with longer distances between them. This indicates that not only do indels and base substitutions tend to co-localize in the same CDS region (as described in the previous section), but tend to co-occur in the same sample as well.

### Evolutionary view of the cancer gene regions in the three human genomes

**Figure 5:**
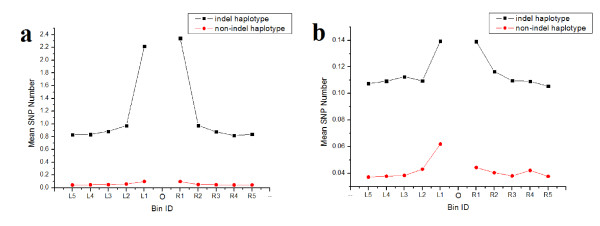
**Evolutionary analysis of SNP distribution in indel flanking regions in cancer genes**. (a) SNP distribution pattern from the alignments of human-Celera, human-Ref, and chimpanzee sequences. (b) SNP distribution pattern from the alignments of human-Korean, human-Ref, and chimpanzee sequences. 'O' denotes indel's position. L1-L5 represents the five bins (100 bp each) in the 5' flanking region, and R1-R5 represents the five bins (100 bp each) in the 3' flanking region. 'Indel haplotype' denotes the SNPs shared with indel haplotype, and 'non-indel haplotype' denotes the SNPs which are not shared with indel haplotype.

Our studies above revealed a close relationship between indels and base substitutions in the CDS of human cancer genes. All of the results above are based on human somatic mutations, or non-evolutionary mutations, which have a tendency to be eliminated in the course of evolution by natural selection. With the advent of new sequencing technologies, more and more human genomes are being sequenced. Here, we used three fully-sequenced human genomes -- human-Ref, human-Celera, and human-Korean (see Methods), with chimpanzee sequences as a reference. By analyzing the single nucleotide polymorphism (SNP) distribution pattern near indels, we found that the SNPs tend to accumulate near the indel in the cancer related genes as well, especially for SNPs linked with the corresponding indel (Fig. 5). There is a slight difference between the pattern in Fig. 5a &5b, presumably due to the fact that only <30 bp indels could be detected under the sequencing method used to obtain the human-Korean genome, resulting in a the relatively smaller dataset than that of human-Celera. All these results were consistent with of our previous publications [24], indicating that indels have a strong influence on a substantial amount of base substitutions in their flanking regions.

## Discussion

### Patterns of somatic mutations

Mutations, which can be categorized into somatic and meiotic ones, play a key role in disease progression and in shaping genomic evolution [30,31]. One of the most distinctive characteristic of somatic cancer mutations is that they are not passed on to the next generation, which renders them unaffected by natural selection. This particular characteristic leads to a distinct pattern between somatic and gametic mutations.

Cancers arise from somatic mutations that confer selective growth advantages on the cells [15]. To understand tumorigenesis, it is essential to define the patterns of somatic mutation, relative frequency of indel and base substitution, and distribution of those mutations. The understanding of the real rate of somatic mutation in normal human cells is still relatively rudimentary [15]. However, mutation rate is likely to differ greatly among different types of cancer cells or genes. For example, in a screen of somatic mutations from 210 cancers, a great difference was found among these cancers [7]. Seventy-three showed no somatic mutations at all, whereas 77 mutations per Mb were present in two gliomas.

The relative low ratio of base substitution to indel in the COSMIC database is interesting. This could represent a true significance of indel mutations in cancer genes or reflect an under-representation of synonymous substitutions in the COSMIC database because the database is designed to catalog mostly missense changes [28]. The underestimated synonymous substitutions could be estimated when a reference database is used. A systematic survey [7] revealed 219 synonymous and 674 non-synonymous substitutions in cancer-related genes, equaling a ratio of 1:3.078 (or 0.3249) of Syn:Non-syn substitutions. In the COSMIC database, we identified 2133 synonymous and 9462 non-synonymous substitutions, or 1:4.436 in Syn:Non-syn substitutions. Based the ratio 0.3249, the total synonymous substitution number should be 3074 (= 9462 × 0.3249) and the total substitutions should be 12536. After this correction, the S/I ratio should be 2.59 (= 12536/2884). Thus, a much lower S/I ratio is still present, compared with the S/I of 11.39 in normal CDS of humans, suggesting a particular importance of indel mutations in driving the occurrence of cancers.

Alternatively, a low S/I ratio could also be caused by the method of mutation screening process. Indeed, some methods, i.e., the protein truncation test, cannot detect mutations that cause missense amino acid substitutions. However, most of the methods identify base substitutions and small insertions/deletions [28], indicating that large indels are often underestimated. Furthermore, the sensitivity of most methods to detect insertions and deletions is low, suggesting the need for additional approaches to their discovery [32]. Be that as it may, it is most likely that indel mutations are very important in cancer genes, and many indels observed might change the functions of oncogenes [8].

In cancer genes, large scales of somatic mutations, either in the form of base substitutions or indels, are highly concentrated towards certain loci in CDS (Table 1, Additional file 1: Supplemental Fig. S3 & S4, and Fig 4). Our previous studies suggested the presence of indels as local mutation enhancers in surrounding sequences in a variety of organisms [24,33] and indicated the non-trivial role indels play in controlling differences of genetic variation and divergence across functional regions of a genome [27]. Compared to meiotic mutations accumulated in the course of evolution, the somatic mutations in cancer genes obviously boasts a much higher correlation of indels and base substitutions in cancer genes. The close correlation between indels and base substitutions at specific regions indicates that those two forms of somatic mutations are essentially associated with the promotion of oncogenesis.

The co-localization of indels and base substitutions in somatic mutations of cancer genes raises an interesting question: how did it occur. The hypothesis that indels induce local mutation during meiosis [24] probably does not apply to somatic tissues because homologous chromosomes do not pair during mitosis. Although there had been reports of pairing of certain chromosomal regions in mammalian cells, the observed frequency was low [34,35]. This co-localization could result from the mutability of specific sequence influenced by its compositional property or its intensity from natural selection. In this case, both types of mutations like to occur simultaneously in response to a region's functional constraint. The uneven distribution of somatic mutations had been observed across the genome in cancer genes [32], however, the data alone cannot validate this hypothesis. Alternatively, this co-localization could be particularly associated with the development of cancer cells, which eventually causes genome instability or their growth advantage. In this scenario, both base substitutions and indels could occur abruptly in large number after a certain stage of cancer cell development. The lower S/I ratio (0.95) in the genes with more somatic mutations (Table 1) indicates that a large number of indels occurred preferentially at the stage when somatic mutation overflows. This hypothesis could be tested by exhaustive detection of both mutation types in the process of cancer cell development in future. The sequencing technologies for such detection are still under development. The generation of thousands of comprehensive and high quality somatic mutations will provide powerful insights into the exact mechanism of co-localization of somatic base substitutions and indels.

### Potential effects of indel mutations on cancer genes

Indel mutations theoretically should be more deleterious than base substitutions, including nonsynonymous changes [27]. Base substitutions have a broad distribution of selective effects, ranging from completely neutral to lethal. Indel mutations, on the other hand, will definitely cause amino acid changes when indel sizes are 3n or trigger frameshift mutations when they are non-3n. Thus, as selective constraint on protein function increases, selective effects of indels and non-synonymous mutations in the same codons or functional regions of a protein are correlated but with indels increasing in their negative selective effects faster than amino acid replacement changes.

It is well-known that there are two kinds of mutations, the 'driver' and 'passenger' mutations [15]. Driver mutations can confer a selective growth advantage and has been positively selected in oncogenesis [7], while the passenger mutations likely have no contribution to the genesis of cancers [15]. A higher ratio of non-synonymous:synonymous somatic mutations than expected by random indicates the presence of positive selection and driver mutations [7]. A genome-wide survey revealed an excess of non-synonymous mutations compared with that expected and thus provided evidence for the existence of driver mutations [7]. Similarly, a higher proportion of 3n indels compared with what is expected by chance (1/3 for example) suggests a positive selection for such size indels. In fact, the expected value should be less than 1/3 because 1-2 bp indels are dominant in cancer genes. As shown in Additional file 1: Supplemental Table S1, 49% of indels of COSMIC database are 1-2 bp ones and 23% are 3n ones. In the 16 tumor suppressor genes with ≥100 mutations, 58% of indels are 1-2 bp ones and 12% of indels are 3n ones. In contrast, the ratio is 3% and 95% respectively for the six oncogenes with ≥100 mutations, where the proportion of 3n indels is 7.9 times larger than that of the tumor suppressor genes (The result is quite similar when using genes with ≥50 mutations). This strongly indicates that 3n indels at some of cancer-related genes are under positive selection and could often be the 'driver mutation'. Therefore it is reasonable to assume that specific driving forces may exist for 3n indels, which could change the function of a gene but not abolish it by frameshift mutation in cancer genes. The particular abundance of indels in the COSMIC database also indicates that indels themselves are positively selected in somatic mutations of cancer genes. In this scenario, more focused studies on indel mutations are especially important for understanding of somatic mutations on oncogenesis.

Studies of various organisms have shown that the occurrence of mutations (particularly indels) is highly influenced by the context and position in the genome [27,31]. In functional sequences, such as coding regions, indels are subject to purifying selection to remove their deleterious effects on gene functions. Different from meiotic mutations, indels have much less selective pressure in somatic mutations, particularly in cancer genes. Given that indels are more likely to be drivers, a substantial number of indels in the CDS of cancer genes are not surprising. For example, 12 genes out of 25 shown in Table 1 have more indels than SNPs in CDS, and such a great proportion of indels in CDS is rarely seen in meiotic mutations. The abundance of indels, which have a comparatively greater influence than SNPs, is consistent with the abnormal function and devastating characteristics of cancer.

### Indel hot-spots in somatic mutations of cancer genes

The mutation pattern of cancer genes demonstrated a distinctive pattern of the indel hot-spots. As shown in Fig. 1a, the indels are not randomly distributed across a gene. They tend to occur at a specific region, e.g., at the 800 - 1078 bp region of the CEBPA gene. There are 114 indels in this region and our analysis also showed that the indel sizes including those in indel hot-spots are far from random (Fig. 1b). Normally, one- or two-bp indels and non-3n indels are dominant in both genome and coding sequences. However, for the 800 - 1078 bp region of the CEBPA gene, 85% are 3n indels. Compared with the first 800 bp region, the 3n indels are 9.44 times more (85% *versus *9%). This great difference indicates that 3n indels at some loci of indel hot-spots are also under strong positive selection.

Unfortunately, little is known about such driving forces. It may vary for different cancer genes, because the strength of co-occurrence between indels and base substitutions is different. Notably, all six oncogenes in Table 1 belong to the 'apparent' category that has a biased mutational pattern. In contrast, the tumor suppressor genes are more likely to have a medium biased or more evenly distributed mutational pattern. This fact suggests that the driving forces, for more 3n bp indels or more concentrated distribution between indels and substitutions, may be associated with oncogenesis itself. This may also shed light on why the strength of co-localization between indel and substitution is so different and why the S/I ratio varies considerably among different cancer genes. The various pattern and S/I ratio among the 25 cancer genes in Table 1 may exhibit the different preference of mutation types in different cancer genes and the diversified underlying mechanisms of cancer.

It is also likely that the driving forces are grounded in a polyclonal epigenetic disruption of stem/progenitor cells, mediated by 'tumor-progenitor genes' [6]. In this scenario, such genes may trigger a spectrum of mutations, which lead to the selective overgrowth of a monoclonal population of tumor cells. To a given gene, the indel hot-spots may be one of a range of possible mutations. Although the detailed mechanisms are not yet clear, indel mutations have apparent important effects on oncogenes.

## Conclusions

All cancers arise from the accumulation of somatic mutations in cancer related genes. This study analyzed the mutational patterns of indels and base substitutions in these genes in the COSMIC database. We found three distinct patterns of indel distribution in somatic mutations: high proportion, great abundance and non-random distribution. A much higher proportion of indels were observed in somatic mutations, as compared to meiotic ones. Also, there is a great diversity of indels at some loci of cancer-related genes. Remarkably in the genes with abundant mutations, the proportion of 3n indels in oncogenes is 7.9 times higher than that in tumor suppressor genes. Considering the dramatic effect of indels on gene function, the non-random distribution of indels, particularly the 3n and non-3n indels, may indicate that indels and their sizes were frequently under positive selection and can often be the 'driver mutations' in oncogenesis.

## Competing interests

The authors declare that they have no competing interests.

## Authors' contributions

DT and JQC designed the study. HY and YZ carried out the sequence alignment and statistical analysis. CP participated in sequence analysis. DT, HY and JQC drafted the manuscript. All authors read and approved the final manuscript.

## Pre-publication history

The pre-publication history for this paper can be accessed here:

http://www.biomedcentral.com/1471-2350/11/128/prepub

## Supplementary Material

Additional file 1 **This file contains all the supplemental figures and tables**. Click here for file
