# Supplementary material for: Important role of indels in somatic mutations of human cancer genes
Source: BMC Med Genet. 2010 Sep 1;11:128. doi: 10.1186/1471-2350-11-128 (PMC2940769; doi:10.1186/1471-2350-11-128)
Supplement: Additional file 1 — This file contains all the supplemental figures and tables. [file 1471-2350-11-128-S1.DOC]

# Supplemental Material

**Supplemental Table S1.**

|  | **Gene  number** | **Indel number ^b^** | | | |
| --- | --- | --- | --- | --- | --- |
|  |  | **All** | **3N** | **Non-3N** | **1-2 bp** |
| **All data in COSMIC ^a^** | 357 | 4370 | 1022 (23%) | 3348 (77%) | 2138 (49%) |
| ***Genes with mutation ≥ 100*** |  |  |  |  |  |
| **Tumor Suppressor Gene** | 16 | 2677 | 309 (12%) | 2368 (88%) | 1552 (58%) |
| **Oncogene** | 6 | 423 | 403 (95%) | 20 (5%) | 11 (3%) |
| **Other** | 3 | 439 | 129 (29%) | 310 (71%) | 149 (34%) |
| ***Genes with mutation ≥ 50*** |  |  |  |  |  |
| **Tumor Suppressor Gene** | 21 | 2839 | 333 (12%) | 2506 (88%) | 1629 (58%) |
| **Oncogene** | 9 | 450 | 421 (94%) | 29 (6%) | 15 (3%) |
| **Other** | 5 | 490 | 130 (27%) | 360 (73%) | 149 (30%) |

1. Genes with at least one indel are used. (b) Frequencies are shown in parentheses.

**Supplemental Fig. S1.** Proportion of indel and base substitution from mutation-rich genes to mutation-poor genes.


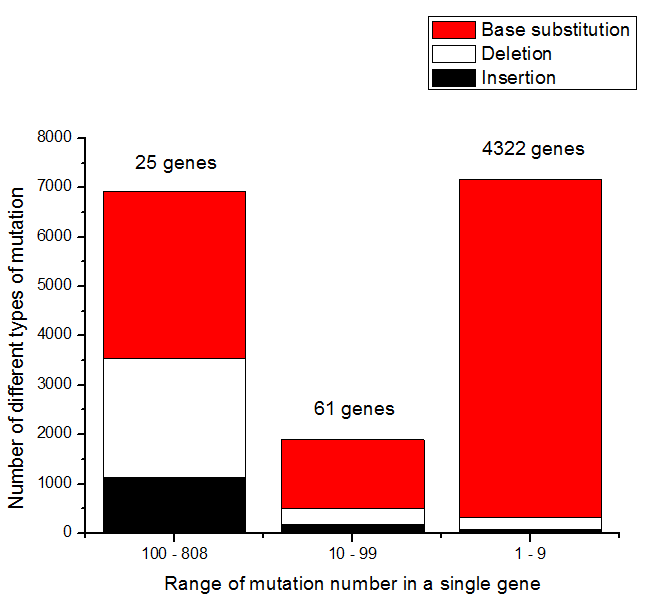


The left column, whose data is same as that in Table 1, contains genes with mutation number from 100 to 808. The middle column contains genes with mutation number from 10 to 99. The right column contains genes with mutation number from 1 to 9. The number above each bar is the number of cancer genes that involved.

**Supplemental Fig. S2.** Indel size distribution of all the data in COSMIC database.


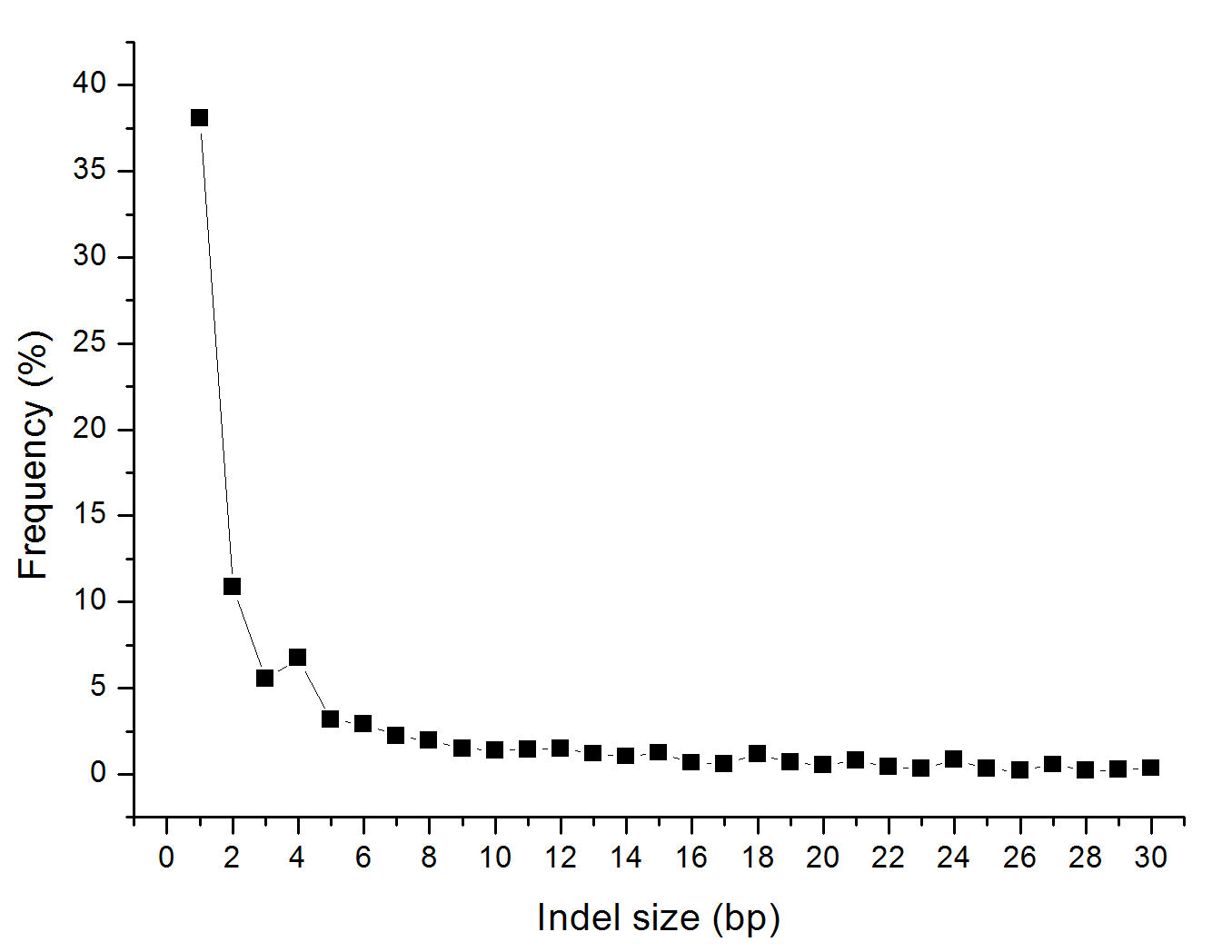


Only ≤ 30 bp indels are shown.

**Supplemental Fig. S3.** Illustration of the eleven genes that belong to the ‘apparent’ category with not less than 100 mutations.


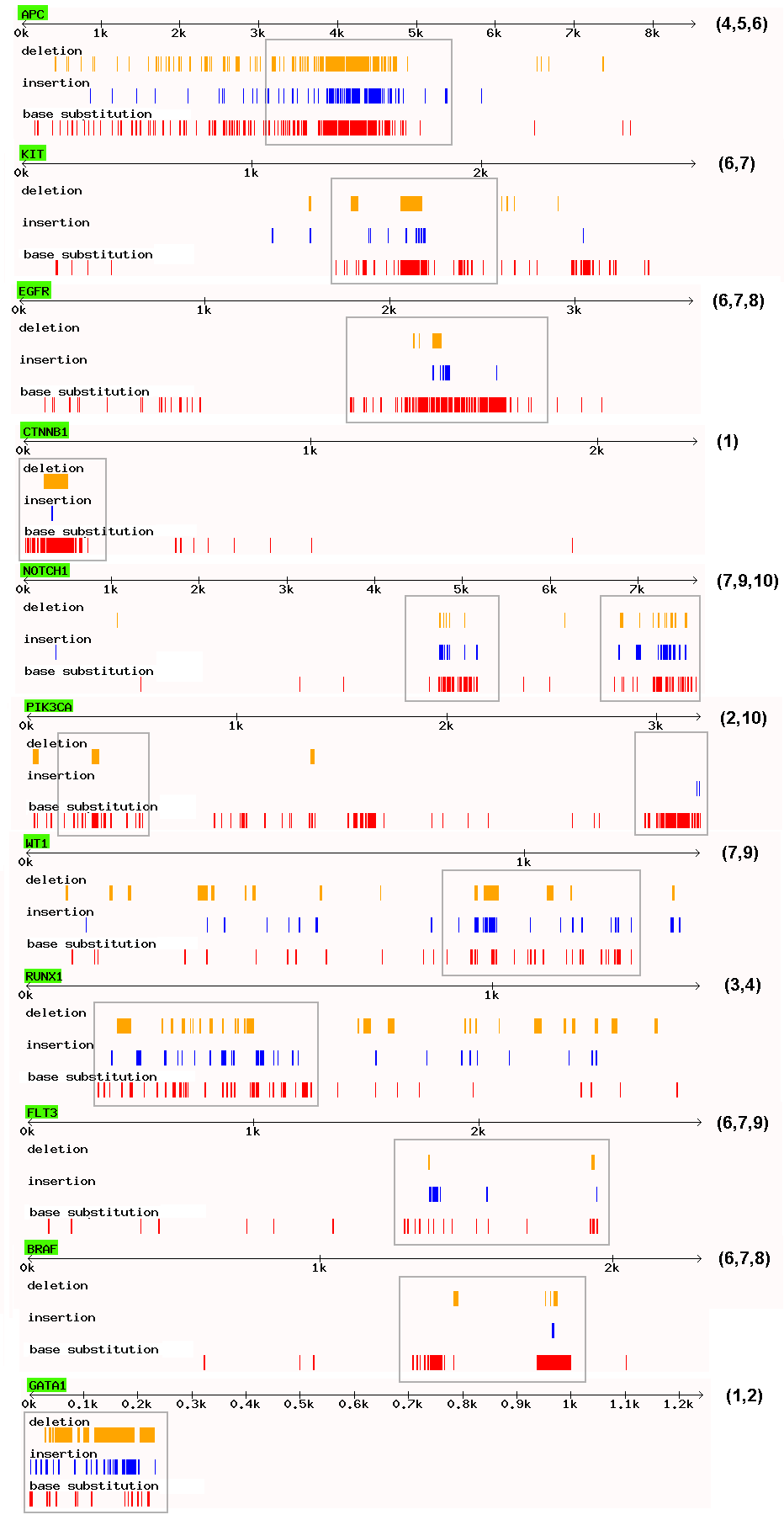


Gene names are shown in green boxes. The Arrow below the gene names represents position in the CDS, and ‘k’ means 1000 bp length of DNA. Each CDS is equally divided into 10 blocks, and the numbers in parentheses on the right side of the arrow is the blocks (meeting the three criteria of ‘apparent’ category in Methods) that are rich in both indels and substitutions. For instance, ‘(2, 10)’ means 10%-20% and 90%-100% of the CDS are mutaton-rich regions for both indels and substitutions. The grey boxes are co-localization regions of indels and subsitutions, drawn with the guide of the blocks in parentheses. To make the indels as less overlap as possible, only ≤ 30 bp indels are shown.

**Supplemental Fig. S4.** Correlation between indel and substitution number for the 25 genes with not less than 100 mutations.


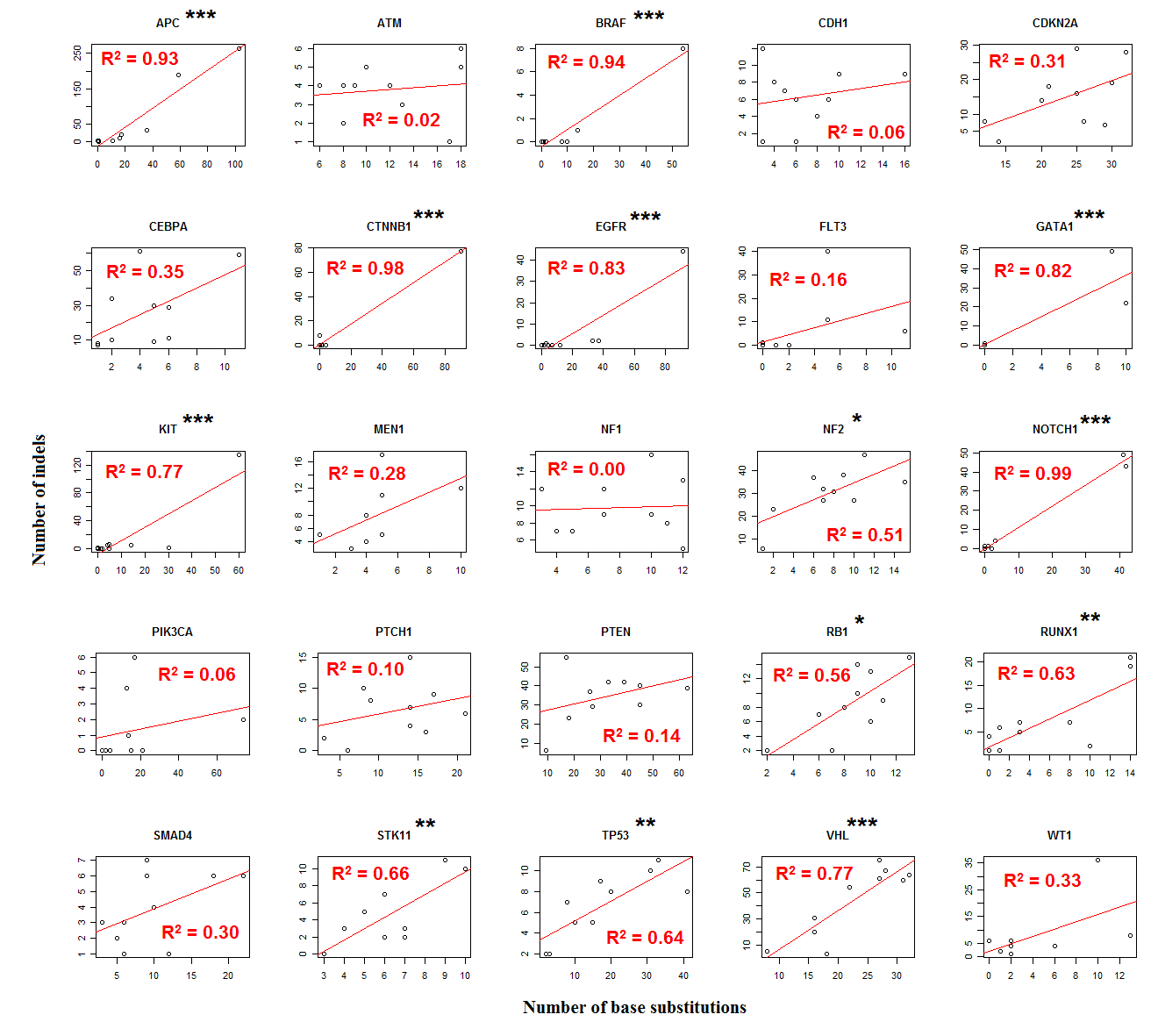


* denotes *P*-value < 0.05; ** denotes *P*-value < 0.01; *** denotes *P*-value < 0.001; Only genes with mutations ≥ 100 are included. For the ‘apparent’ genes (i.e. CTNNB1), data is missed in some blocks of CDS, leading to a biased distribution pattern, high R-squre, and low *P*-value.

**Supplemental Fig. S5.** Illustration of insignificant correlation between indel and base substitution in ten-block analysis. (a) Number of mutations in ten sequential blocks of NF1; (b) scatter plot of indels and base substitutions in NF1; Graphic view of mutations in NF1 is in Fig. 2c.


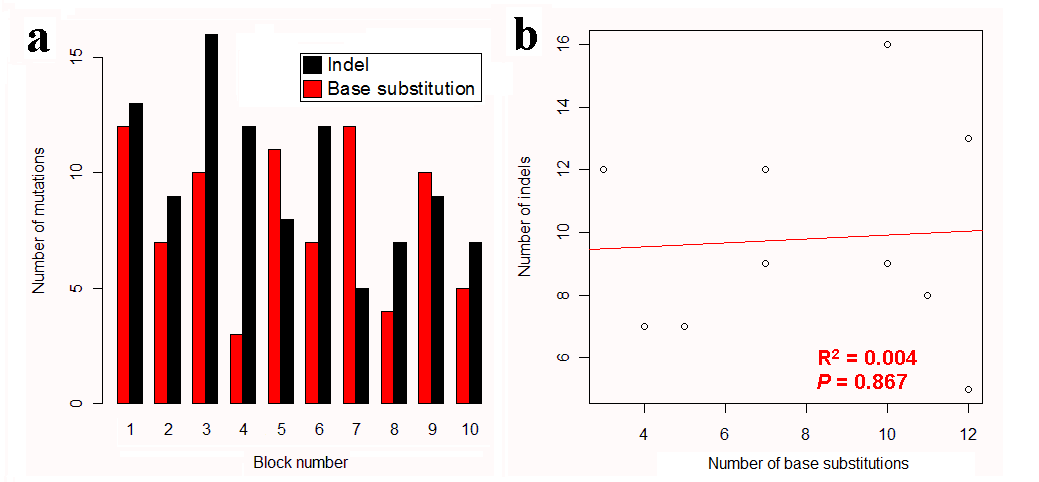


**Supplemental Fig. S6.** Illustration of the ten genes with mutations from 50 to 99.


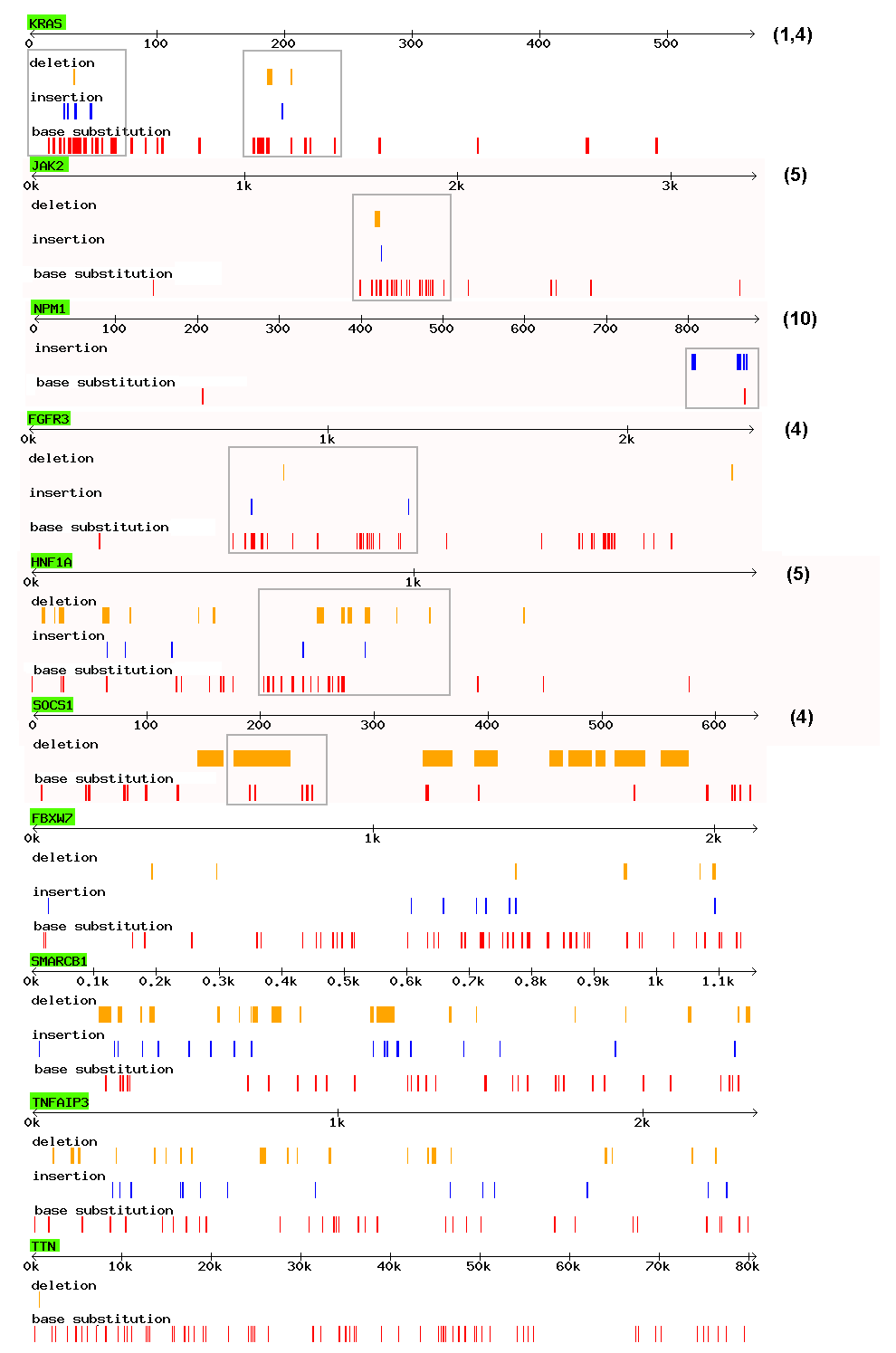


Gene names are in green boxes. To make the indels as less overlap as possible, only ≤ 30 bp indels are shown. For 10 genes with ≥ 50 mutations and ≤ 99 mutations, six genes (KRAS, JAK2, NPM1, FGFR3, HNF1A, and SOCS1) reach the ‘apparent’ criteria (the bracket and grey box have the same meaning as that in Supplemental Fig. 3). When plotting indels against substitutions in 10 blocks, KRAS, NPM1, JAK2 reach the threshold R^2^ > 0.40 and *P* < 0.05, and FBXW7 (R^2^ = 0.33 and *P* = 0.08) is close to the threshold. After all, the threshold (of ‘apparent’ and ‘significant’) is made to explore genes with ≥ 100 mutations; thus it may be not that suitable to perfectly work on genes with less mutations. But still, at least 30% - 60% of the ten genes manifest the co-localization, indicating the validity of our main results.
